# Characteristic values of the lumbar load of manual patient handling for the application in workers' compensation procedures

**DOI:** 10.1186/1745-6673-6-17

**Published:** 2011-05-26

**Authors:** Claus Jordan, Alwin Luttmann, Andreas Theilmeier, Stefan Kuhn, Norbert Wortmann, Matthias Jäger

**Affiliations:** 1 Leibniz Research Centre for Working Environment and Human Factors (IfADo) Ardeystr. 67, 44139 Dortmund, Germany; 2 German Aerospace Center, Project Management Agency, Heinrich-Konen-Str. 1, 53227 Bonn, Germany; 3 BGW - Institution for Statutory Accident Insurance and Prevention in the Health and Welfare Services, Göttelmannstr. 3, 55130 Mainz, Germany; 4 BGW - Institution for Statutory Accident Insurance and Prevention in the Health and Welfare Services, Pappelallee 35/37, 22089 Hamburg, Germany

## Abstract

**Background:**

The human spine is often exposed to mechanical load in vocational activities especially in combination with lifting, carrying and positioning of heavy objects. This also applies in particular to nursing activities with manual patient handling. In the present study a detailed investigation on the load of the lumbar spine during manual patient handling was performed.

**Methods:**

For a total of 13 presumably endangering activities with transferring a patient, the body movements performed by healthcare workers were recorded and the exerted action forces were determined with regard to magnitude, direction and lateral distribution in the time course with a "measuring bed", a "measuring chair" and a "measuring floor". By the application of biomechanical model calculations the load on the lowest intervertebral disc of the lumbar spine (L5-S1) was determined considering the posture and action force data for every manual patient handling.

**Results:**

The results of the investigations reveal the occurrence of high lumbar load during manual patient handling activities, especially in those cases, where awkward postures of the healthcare worker are combined with high action forces caused by the patient's mass. These findings were compared to suitable issues of corresponding investigations provided in the literature. Furthermore measurement-based characteristic values of lumbar load were derived for the use in statement procedures concerning the disease no. 2108 of the German list of occupational diseases.

**Conclusions:**

To protect healthcare workers from mechanical overload and the risk of developing a disc-related disease, prevention measures should be compiled. Such measures could include the application of "back-fairer" nursing techniques and the use of "technical" and" small aids" to reduce the lumbar load during manual patient handling. Further studies, concerning these aspects, are necessary.

## Background

Diseases at the muscle and skeleton systems belong to the most frequent causes for health-related absenteeism in the workplace. Handling heavy objects increases the risk of low back pain. This is also a significant problem among nurses [1] because care-activities with manual patient handling may lead to high load on the spine [2,3] and may accelerate the development of degenerative disc-related diseases in the long run of the occupational life [4,5].

In Germany, the social protection of the inhabitants is based to a big part on a statutory insurance system, the social insurance (Sozialversicherung). The statutory social insurance consists of the compulsory health insurance, the long-term care insurance, the pension insurance and, particularly regarding the problem discussed here, the statutory accident insurance. Supporting organisation of this statutory accident insurance for the enterprises of the German business companies and their employees are the Statutory Accident and Health Insurance Institutions. Their commission is to avert and, in case, to compensate for occupational accidents and diseases. Employees which have suffered from an occupational accident or suffer from an occupational disease are rehabilitated by the Statutory Accident and Health Insurances medically, occupationally and socially. In addition, the consequences of accidents and diseases are financially compensated for. Mainly diseases which are listed in the Occupational Diseases Regulation (Berufskrankheiten-Verordnung/BKV) can be admitted. The responsible statutory accident and health insurance institution accomplishes an occupational disease evaluation where criteria for the relationship between a possibly damaging effect of the occupational activity and the diagnosed disease are checked. For that purpose a retrospective determination and evaluation of the lifetime occupational exposure is necessary. If this association is found and the damage is confirmed medically, an occupational disease is admitted. In the context of degenerative diseases in the lower-back region, as for example intervertebral disc-related diseases of the lumbar spine caused by long-term lifting or carrying heavy objects or caused by long-term activities in extremely trunk-flexed postures, were enacted in the Occupational Disease Regulation relatively newly as the occupational disease OD 2108 (Berufskrankheit BK 2108) [6].

For the retrospective load analysis the so-called Mainz-Dortmund Dose Model ("MDD") [7,8] is used regularly in Germany. The biomechanical low-back load is considered by its amount per relevant single action - represented by the action-specific peak compression force on the lumbosacral disc - as well as its frequency of occurrence and duration, and evaluated concerning its cumulative impact regarding the biomechanical risk of overload of the lumbar spine. The result is a cumulative exposure measure in form of the day-related assessment-dose for typical shifts ("daily dose") and the cumulated dose for the total vocational life-span ("lifetime dose"). The MDD is easily applicable for the retrospective analysis of conventional lifting, carrying and holding-of-object actions. For nursing activities with manual patient handlings, however, more detailed knowledge was necessary, because these actions differ in various regards from "usual" lifting and carrying procedures, i.e. the application of the MDD had to be modified. On the one hand, knowledge of patient's body weight only is not sufficient, because with a patient-handling action, normally not the whole body is raised. On the other hand, the patient is commonly not so much lifted as rather transferred horizontally and, because of the intended positioning task, the exertion of caregiver's forces underlies a great variance due to, inter alia, the different transfer-techniques used by the healthcare workers. In 2001 the Statutory Accident and Health Insurance Institution for Health Services and Welfare Care (Berufsgenossenschaft für Gesundheitsdienst und Wohlfahrtspflege / BGW) developed a preliminary procedure for dose calculation in order to define the operational requirements of occupational-disease statement procedures for the analysis of healthcare activities [9]. Based on simplifying assumptions such as a standardised average patient-weight and an unspecified handling technique lumbar load was estimated for relevant transfer activities. These estimated characteristic values of the lumbar load had to be questioned and supplemented by objective measurements.

The research project introduced here - the so-called Dortmund Lumbar Load Study 3 ("DOLLY 3") [10] - was carried out in collaboration with the BGW. DOLLY 3 was aimed for to determine quantitatively the load on the lumbar spine for typical manual patient handlings (e.g. raising a patient from a lying position to a sitting posture in bed) and to derive characteristic values of lumbar load which can be used in occupational-disease statement procedures concerning the OD 2108.

## Methods

The underlying methodology is described within three main parts. The first part overviews the adopted biomechanical simulation and evaluation tool used in this study, and the second part describes the experimental procedure applied to determine the load of the lumbar spine of healthcare workers during manual patient handling. The last part introduces the scope of investigated transfer actions. The examinations were not performed in a hospital but in the laboratory due to applying a complex measurement-assisted methodology for the determination of lumbar load based on posture-and-force capturing. For ethical and also technical reasons no real patients served as subjects. Instead, two professionally experienced female healthcare workers acted alternately as a patient or a nurse throughout the research project. They are both highly qualified in applying different measuring variables like fully versus partially co-operating patient, i.e. to give more or less support during co-operating with the carer. In this context the patient was, at least, partially co-operating and the task was executed by the healthcare worker as commonly performed in hospitals. That means the handling of totally non co-operating patients was not studied explicitly.

### Biomechanical modelling

Several measures of lumbar load were quantified by means of inverse dynamics on the basis of measured posture and action-force data via model calculations. To this end a previously developed simulation and evaluation tool, "The Dortmunder", was applied [11,12]. This validated tool bases upon a 3-D multi-segmental dynamic biomechanical model of the relevant human skeletal and muscular structures. It allows the quantification of various low-back load indicators considering gravitational and inertial effects of the body and a potentially handled object - here the subject "patient" - and in particular, effects of asymmetry regarding posture and force exertion. The human skeletal structure is represented by 30 body segments which are considered as mechanically rigid bodies and supported in 27 punctiform joints in total. Each body segment, supposing a cylindrical shape, is characterized by its length, radius and distance between the centre of gravity and the adjacent joint, its weight as well as the moment of inertia. The intervertebral discs within the trunk up to shoulder height - i.e. five lumbar discs and the lower ten out of the twelve thoracic discs - are considered as joints. Consequently, sagittal and lateral bending, twisting as well as the superposition of both flexion and torsion of the trunk can be replicated realistically.

The muscular structure in the lower trunk region, spreading over the lumbar discs and connecting pelvis and rib cage biomechanically, is simulated by the effect of 14 muscles or muscle cords at the back and the abdominal wall. The back musculature, summarized in the Erector Spinae muscle group, is represented by its two main cords: the Longissimus muscle with its lumbar part and the Iliocostalis muscle with its medial part which are implemented each on both sides of the body; these muscle cords are modelled by four equivalent force vectors. The functional behaviour of the anatomically fan-like shaped Abdominal Oblique muscles is also considered in the model: The medial muscle cords of the internal and external parts of opposite sides are connected via a tendinous network which particularly enables twisting the trunk; in contrast, the lateral muscle cords are mainly activated during side-bending postures. Consequently, the muscles cords of the Abdominal Obliques are replicated by other four equivalent force vectors. The two cords of the Rectus Abdominal muscle are located beneath the tendinous texture mentioned above and are running parallel near the mid-sagittal plane; as a result, a single force vector only is considered in the model and acting as an antagonist of the back muscles mainly in sagittal procedures. Hence nine force vectors simulate the effect of fourteen muscle cords in the lower trunk region in total.

### Experimental procedure

**Figure 1:**
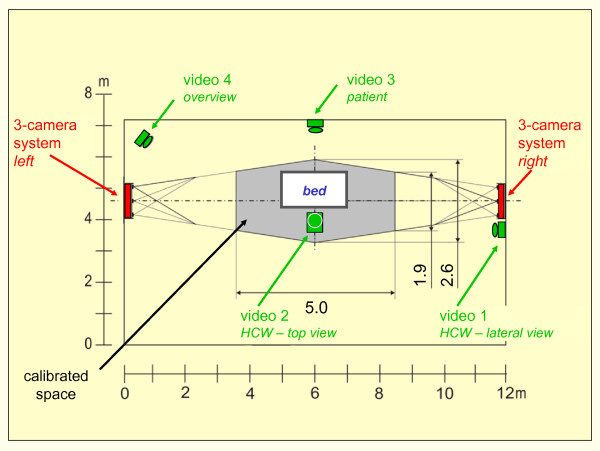
**Schematic representation of the experimental setup**. Ground view of the laboratory with measuring bed, two combined OPTOTRAK 3-camera systems forming the calibrated measuring space and positioning of four video cameras, enabling the documentation of posture and movement of the healthcare worker (HCW) during a manual patient handling activity (for detail see text).

Analaysis of a manual patient handling action assumes the information of two important variables: the knowledge on the action forces exerted and on the postures adopted by the healthcare worker. Knowledge of the posture was achieved by using a combination of videoanalysis and optoelectronic measurement [13]. The video recordings were accomplished by two cameras: one was installed beside the healthcare worker to document preferably the trunk's forward inclination and spinal curvature in a lateral view (video 1 in Figure 1), the 2nd camera above the healthcare worker was mounted at the ceiling recording a top view indicating sideward bending and turning in main (video 2 in Figure 1). Applying a split-screen technique representing both video frames simultaneously on a single monitor, a synchronous analysis of both views was guaranteed. Patient's posture and movement was documented via a 3rd camera, whereas a 4th one was used to receive a spatial view of the scene.

For the optoelectronic measuring, a 3-D motion and position measurement system "OPTOTRAK" (NDI, Northern Digital Inc., type 3020) was applied, which tracks small infrared markers attached to the subject at relevant anatomical landmarks. Markers were attached to the shoulders, the hands, the hip joints and the heels of the healthcare worker. These body parts were chosen because of their importance for the lumbar-load level and, correspondingly, the biomechanical model calculations. Additionally two markers were applied to the bed posts at the long side of the bed - or the chair or the floor - as a reference.

Two "position sensors", as the main components of the system consisting of three infrared cameras each which are arranged in a firm angle and distance to each other, are required to determine the 3-D position of each marker. These position sensors were mounted at opposite walls of the laboratory; their visual fields overlap and form a calibrated space in the middle of the room surrounding the measuring bed and the healthcare worker (see Figure 1). The contralateral positioning of the sensors enables capturing of the marker localisation at both sides of the observed person.

The system OPTOTRAK determined the three-dimensional inertial co-ordinates of the markers continuously over the entire handling time. These local positions with reference to the laboratory were then transformed into the co-ordinate system of the healthcare worker replicated in the simulation tool The Dortmunder. The subsequent digital reproduction of the actual posture of the observed healthcare worker consisted of two steps: In a first phase the posture was described roughly by a stick figure on the basis of the video photographs with the help of the specially developed graphically supported input system. In a second step, for the accurate reproduction of the posture, the respective co-ordinates of the modelled body segments were set into coincidence with the co-ordinates of the markers at the caregiver's body. This iterative procedure was necessary to replicate the posture as realistic as possible, even in cases, when a marker is covered. For example, the marker at a hand was hidden when the caregiver grasped under the upper body of the patient to raise her up.

The exerted forces of the healthcare worker during a manual patient handling in the bed were determined with regard to magnitude, direction and bilateral distribution by using a newly developed "measuring bed" [14,15]. For that reason, a common hospital bed was modified and equipped with an additional framework, which was inserted between the bedstead and - via tri-axial force sensors at the four corners - the bedspring frame. That enables an "indirect" measurement of the forces of the healthcare worker in three components (upward, forward, sideward or vice versa). The point of application of the resultant hand-force was derived from bed-forces' distribution. Leaning against the bed was considered via an additional sensor-equipped bar at the bed's side. Two force platforms (Kistler, type 9281B13) were used for ground-reaction force recording at the floor in cases when the patient was leaving the bed.

In order to examine transfer activities like "Placing a patient from sitting at bed's edge in a chair and vice versa", a measuring system "chair" was developed on the basis of a commonly used toilet-chair mounted on a force plate. The action forces of the healthcare worker were then derived from the signals of the four force sensors in the force platform. The height of the measuring chair could be adapted according to the requirements of the specific patient handling. Furthermore, footstep-bridges were positioned above the platform avoiding a contact of the healthcare worker with the measuring system and to separate patient-induced from nurse's ground-reaction forces. Analogously a "measuring floor" was configured applying two force platforms simultaneously, which enabled force recording during transfers such as "Raising a lying patient from floor".

On the basis of the combined data of posture, exerted forces, point of force application and individual somatometric parameters, forces and moments of force at the lowest disc of the spinal column were computed applying the biomechanical model The Dortmunder. In this way various lumbar load indicators - such as compressive and shear forces as well as bending and twisting moments with respect to the lumbosacral disc - were determined for several manual patient handlings.

### Scope of analysed tasks

**Figure 2:**
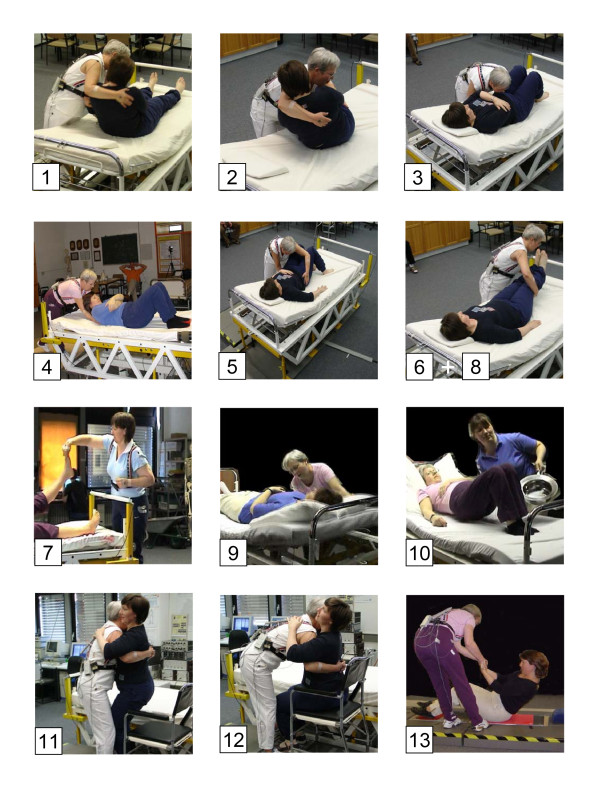
**Representative photos for various patient handlings**. Typical postures of the caregiver and patient for the 13 studied manual patient handling activities: 1. Raising a patient from lying to sitting in bed or vice versa 2. Elevating a patient from lying to sitting at the bed's edge or vice versa 3. Moving a patient towards the bed's head (nurse at bed's long side) 4. Moving a patient towards the bed's head (nurse at bed's head) 5. Moving a patient in the bed sidewards 6. Lifting a leg of a lying patient or vice versa (nurse at bed's long side) 7. Lifting a leg of a lying patient or vice versa (nurse at bed's foot) 8. Lifting both legs of a lying patient or vice versa (nurse at bed's long side) 9. Inclining the bed's head with the patient lying in the bed 10. Shoving a bedpan or vice versa 11. Placing a patient from sitting at bed's edge in a chair or vice versa 12. Raising a patient from sitting to upright standing position or vice versa 13. Raising a patient from lying on the floor to standing position

Various manual patient handlings within the bed, from bed to a chair and vice versa and from the floor to a sitting or standing posture were analysed. The chosen activities are classified by the Statutory Accident and Health Insurance Institution for Health Services and Welfare Care as "definitely being endangering" in the sense of the corresponding occupational disease no. 2108 [16]. Thus the following activities were examined in detail (see also Figure 2):

1. Raising a patient from lying to sitting in bed or vice versa

2. Elevating a patient from lying to sitting at the bed's edge or vice versa

3. Moving a patient towards the bed's head (nurse at bed's long side)

4. Moving a patient towards the bed's head (nurse at bed's head)

5. Moving a patient in the bed sidewards

6. Lifting a leg of a lying patient or vice versa (nurse at bed's long side)

7. Lifting a leg of a lying patient or vice versa (nurse at bed's foot)

8. Lifting both legs of a lying patient or vice versa (nurse at bed's long side)

9. Inclining the bed's head with the patient lying in the bed

10. Shoving a bedpan or vice versa

11. Placing a patient from sitting at bed's edge in a chair or vice versa

12. Raising a patient from sitting to upright standing position or vice versa

13. Raising a patient from lying on the floor to standing position

The photos of Figure 2 give an impression of the listed transfer activities. The numbering of the photos comply with the numbers of the list. Most of the examined movements were also accomplished in reverse direction. The manual patient handlings were performed in a conventional way, that means in a way as it is done in every day life in the clinics. Taking into consideration the number of the listed activities and the before mentioned measuring variables (2 subjects, 2 co-operating levels and 2 positioning directions), about 90 different variations were investigated. The activities were performed in most cases 5 times each to enable the detection of intra-individual execution variations. Each activity was divided into separate sections for the evaluation, in consequence, more than 400 activity phases were analysed. To accomplish the data evaluation, typical executions were selected and a detailed analysis was carried out including the calculations for the diverse lumbar-load indicators. A complete evaluation of all recorded actions had to be renounced due to the enormous and therefore unrealistic additional expenditure of necessary time. In order to check the reproducibility of the measurements, all 18 executions for a typical activity were evaluated, i.e. lifting a leg of a partially co-operating patient lying in the bed or vice versa [17].

## Results

### Typical time courses for lumbar-load determination

### Postures

**Figure 3:**
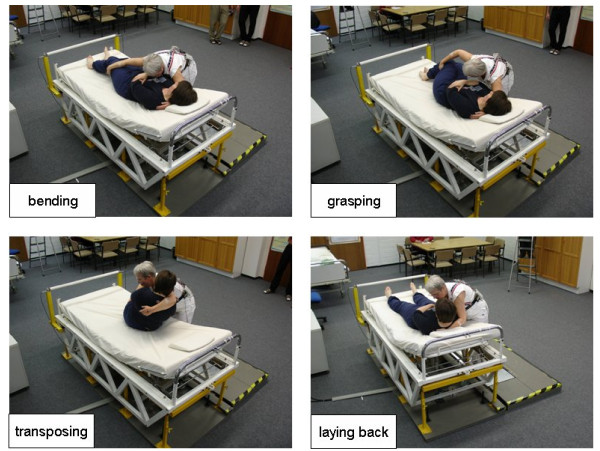
**Representative photos for a single patient handling**. Typical postures of the caregiver and patient for the four phases "bending", "grasping", "transposing" and "laying back" during the manual handling activity "Elevating a patient from lying to sitting at the bed's edge or vice versa" (no. 2 of the list in figure 2).

The exemplarily described activity, elevating a patient from lying in the bed to a sitting position at the bed's edge and vice versa, was divided into sequential segments which were denoted as basic posture, bending, grasping the upper part of the body of the patient, transposing the upper body of the patient, holding the patient, laying back the patient, straighten up and basic posture again. In this context, Figure 3 shows photos of selected situations, which are accountable for relatively high values of the resulting lumbar load, i.e. bending, grasping, transposing and laying back.

The total procedure starts with the healthcare worker just standing at the bedside and waiting for the signal announcing the start of the measurement. Thereupon she was bending forward to the patient in the bed. In detail, caregiver's trunk was flexed forward considerably and turned a little to the left side. The left arm was strongly bent in the elbow joint, the right arm was almost straightened. This posture was also maintained while grasping the upper body of the patient by putting her left arm underneath patient's shoulder and grasping patient's legs at the knee joints with her right arm. After transposing the upper body from a horizontal to an upright position, the patient was stabilised in a constant posture while sitting at the bed's edge with hanging lower legs. After a short phase of holding the sitting patient, patient's upper body was laid back on the mattress to the left side combined with swaying the legs upwards. The healthcare worker bent her own upper body strongly forward and at the same time to the left, both arms were bent in the elbows. After finishing the transfer action, the healthcare worker re-straightened up.

### Action forces at the hands

**Figure 4:**
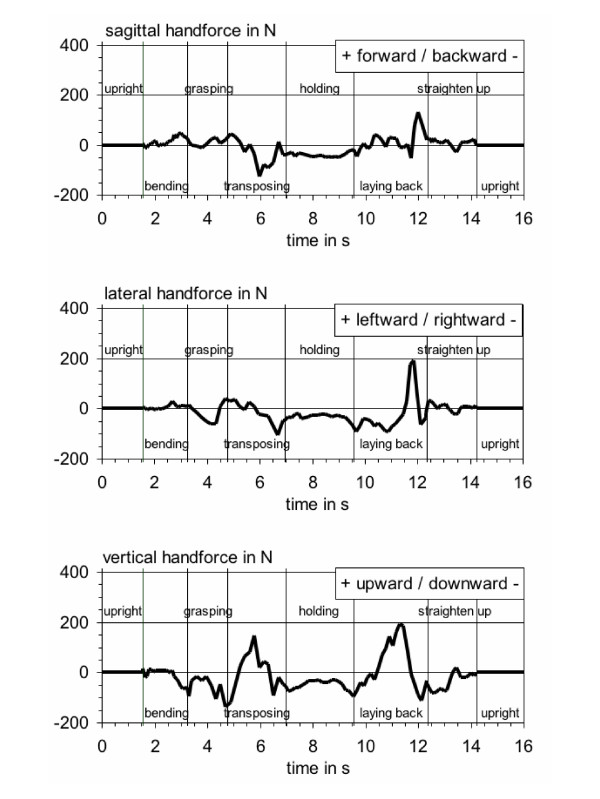
**Action forces at the hands**. Time courses of the components of the action forces at the hands, determined during the activity "Elevating a patient from lying to sitting at the bed's edge or vice versa".

The forces which are transferred from the healthcare worker to the patient during an activity's execution represent the "action forces" at the hands. For the exemplarily chosen activity, the temporal courses of the recorded horizontal and vertical action-force components are shown in Figure 4 in three traces (forward/backward, leftward/rightward, upward/downward).

As mentioned in the subchapter "postures" the relatively complex motion sequence was divided into eight sequential phases. The first noticeable load-relevant segment is the third one, i.e. grasping the upper part of the body of the patient. The temporal courses of the hand-action forces reach a peak value of the force component in downward direction, i.e. supporting caregivers upper body, of nearly -140 N applied with her left arm to patient's shoulder (lower trace) and a component "to the right" of -60 N (middle trace). In the following phase "transposing the upper part of the body of the patient", the direction of the vertical force component was inverted (lower trace) and reached a value of nearly +150 N upwards due to elevating patient's trunk from a lying to a sitting position. Immediately after this local maximum, the action force "backward" reached its peak value with an amount of nearly -130 N (upper trace), resulting from pulling patient's leg from bed's midline to bed's edge. At the end of the transposing procedure, forces of about -100 N each, were exerted by the healthcare worker in the directions "downward" and "to the right", respectively, due to pushing the legs downward accompanied by pushing patient's trunk sideward into an upright position. The clearly highest action-force values, however, were determined for the segment "laying back the patient". In this period four successive peaks in the different traces of the hand-action force components can be identified: The first and second local maximum appear in the vertical component with nearly +140 N and +200 N, respectively (10.8 s / 11.4 s, lower trace); the first load maximum is traced back to swaying patient's hanging legs upwards ("lifting"), and the second one is caused by both the aforementioned leg-lifting action and the holding of patient's trunk against gravity during the laying-back action. These actions are followed by two pushing-the-legs actions directed horizontally, first of all pointing leftward to the bed's head and then forward to the bed's middle axis. Seen from the carer's point of view, the third action-force peak of this transposing section resulted in "leftward" direction (+200 N at 11.8 s, middle trace), and the fourth local maximum of +140 N is shown in the course for forward pushing (12.1 s, upper trace).

### Reaction forces at the lumbosacral disc

**Figure 5:**
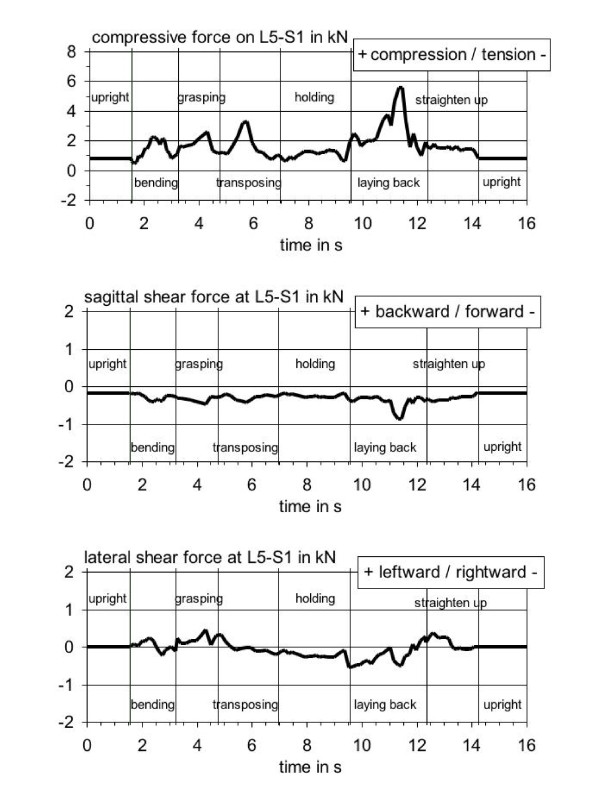
**Forces at the lumbosacral disc L5-S1**. Time courses of the components of the forces at the lumbosacral disc L5-S1, determined during the activity "Elevating a patient from lying to sitting at the bed's edge or vice versa."

In analogy to the courses of the hand-action forces in Figure 4, the highest values of the compressive force on the lumbosacral disc (see Figure 5, upper trace) appear while transposing the upper body of the patient (3.3 kN) and laying back the patient (5.5 kN). Also in the segments "bending" and "grasping the upper part of the body of the patient" the compressive force reached increased values (max. 2.2 kN at 2.3 s or 2.6 kN at 4.2 s). During bending, the exerted action forces are almost zero so that the local compressive-force peak is solely a consequence of the unfavourable posture of the healthcare worker: trunk slightly bent forward and turned sidewards with the arms held frontally. At the time of the local peak in the grasping phase (4.2 s), the disadvantageous posture is superimposed by a relatively high lateral action force (-60 N, i.e. to the right, cf. Figure 4, middle trace). Nevertheless, the resulting disc-compressive force reaches a maximum of "only" 2.6 kN, as the carer leans against the patient at this time causing a partial supporting effect for the trunk (-100 N, i.e. downwards, cf. Figure 4, lower trace at 4.2 s). The highest compressive forces shown in Figure 5 are to be found during transposing and laying back the patient; they are mainly induced by the strong upward hand-forces in these periods (+150 N at 5.8 s and +200 N at 11.4 s, cf. Figure 4, lower trace).

The lumbosacral sagittal shear force reaches its extreme value of about -0.9 kN at laying back the patient (cf. Figure 5, middle trace, at 11.4 s). This can be traced back to the fact that a high vertical action force (+200 N, cf. Figure 4, lower trace) is exerted to lift patient's legs from a hanging position to mattress level and to hold the trunk against gravity in order to avoid a too rapid motion during downward swaying. The highest lateral shear forces at the lumbosacral disc were adopted during the pre-positioning phase "grasping" and during the laying-back action (cf. Figure 5, lower trace). During the way-there action, the relatively high shear force (up to 0.4 kN leftward at 4.2 s) results from grasping patient's upper body at the shoulder and exerting action forces pointing to the right. While laying back the patient, the local maximum of -0.5 kN (at 11.4 s) is caused by an asymmetric posture with the trunk flexed to the left; the exerted lateral action-force components are negligible at this point in time (cf. Figure 4, middle trace at 11.4 s).

### Lumbar load for analysed tasks

**Figure 6:**
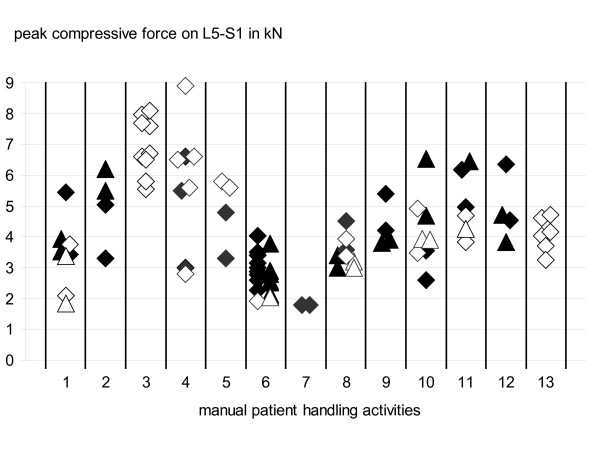
**Lumbar load for various patient handling activities**. Concluding representation of the peak values of the compressive force on the lumbosacral disc L5-S1 for 13 manual patient-handling activities: 1. Raising a patient from lying to sitting in bed or vice versa 2. Elevating a patient from lying to sitting at the bed's edge or vice versa 3. Moving a patient towards the bed's head (nurse at bed's long side) 4. Moving a patient towards the bed's head (nurse at bed's head) 5. Moving a patient in the bed sidewards 6. Lifting a leg of a lying patient or vice versa (nurse at bed's long side) 7. Lifting a leg of a lying patient or vice versa (nurse at bed's foot) 8. Lifting both legs of a lying patient or vice versa (nurse at bed's long side) 9. Inclining the bed's head with the patient lying in the bed 10. Shoving a bedpan or vice versa 11. Placing a patient from sitting at bed's edge in a chair or vice versa 12. Raising a patient from sitting to upright standing position or vice versa 13. Raising a patient from lying on the floor to standing position dark symbol = partially co-operating patient light symbol = fully co-operating patient rhombus = forward movement triangle = backward movement

With respect to lumbar load of the healthcare worker, about 90 representative transfers, i.e. actions being typical regarding posture and motion as well as regarding hand-force exertion, were analysed in total. In Figure 6 the lumbar load is summarised indicated by the peak values read from the corresponding time courses for lumbosacral compressive force for the different manual patient handlings. Most of the activity types are represented by pairs of values, according to the "main" forward direction or the way back, due to the fact that a biomechanical difference of both operations could not to be excluded first of all. In the diagram of Figure 6 these two maxima are distinguished by the form of the symbol (rhombus = way there; triangle = way back). For activities without a return movement (e.g. moving a patient towards the bed's head), merely the results of the way there are given. Another differentiation can be attached to the mobility degree of the patient: Task execution with a fully co-operating patient is represented by an open symbol, whereas closed symbols show the results for partially co-operating patients.

Altogether the diagram shows peak values between approximately 2 and 9 kN concerning the compressive force on the lumbosacral disc of the healthcare worker. Within this large span the lowest values were reached for raising a leg of the patient with the caregiver positioned at the bed's foot (no. 7) whereas the highest compressive forces were reached for moving a patient towards the bed's head (no. 3). In most cases, higher values were found for positioning a more passive patient than moving a more active patient (compare closed vs. open symbols). Furthermore the diagram shows that there is no explicit evidence whether the way there or the way back leads to higher lumbar load. For instance, for "Inclining a bed's head with the patient" (no. 9) higher values were found with the way there than with the way back (rhombi vs. triangles), while for "Elevating a patient from lying to sitting at the bed's edge" (no. 2) the back way lead to higher values.

An essential purpose of the study introduced here was to examine the load of the lumbar spine occurring with manual patient handlings to enable a scientifically supported derivation of characteristic values for lumbar load to be applied in occupational-disease statement procedures concerning the association between biomechanical load of the lower back through manual patient handling and the risk for developing degenerative diseases like disc narrowing and herniation (in Germany, occupational disease no. 2108). The respective list of activities with the corresponding characteristic lumbar-load values are presented in table 1.

**Table 1:** Recommendations for lumbar load assessment procedures

**no**.	activity	Recommendation Compressive force in kN
1	Raising a patient from lying to sitting in bed or vice versa	4.1

2	elevating a patient from lying to sitting at the bed's edge or vice versa	5.1

3	moving a patient towards the bed's head (nurse at bed's long side)	7.0

4	moving a patient towards the bed's head (nurse at bed's head)	6.0

5	moving a patient in the bed sideward	5.0

6	lifting a leg of a lying patient or vice versa (nurse at bed's long side)	2.9

7	lifting a leg of a lying patient or vice versa (nurse at bed's foot)	1.8

8	lifting both legs of a lying patient or vice versa (nurse at bed's long side)	3.7

9	inclining the bed's head with the patient lying in the bed	4.4

10	shoving a bedpan or vice versa	4.6

11	placing a patient from sitting at bed's edge in a chair or vice versa	5.9

12	raising a patient from sitting to upright standing position or vice versa	4.9

13	raising a patient from lying on the floor to standing position	4.1

Recommendations for characteristic values of the lumbar load to be applied in occupational-disease statement procedures concerning the Occupational Disease no. 2108 of the German list of occupational diseases.

The values provided in table 1 mainly considered activities with partially co-operating patients, that means, the results for fully co-operating patients were neglected in order not to underestimate the resulting biomechanical load and the corresponding overload risk for the lower back of healthcare workers. Though, in some cases the carrying out of the manual handling task with an only partially co-operating patient was not possible without taking the risk of considerable biomechanical overload for the caregiver (e.g. moving a patient towards the bed's head). In such cases the measurements were performed with a fully co-operating patient only to protect the subject and, in consequence, the respective values for a more active patient were appointed in the table.

## Discussion

This paper presents characteristic values of the lumbar load of healthcare workers for typical activities with manual patient handling. The collection of data made a very complex procedure necessary. For this purpose measuring systems were implemented to determine the main influencing factors on lumbar load, in particular the posture of the healthcare worker and the forces brought forward to the patient. This information regarding posture and action force was therefore also necessary to carry out the biomechanical simulation calculations with the help of the three-dimensional dynamic simulation tool The Dortmunder to determine characteristic values of the task-induced lumbar load. A comprehensive measuring configuration, consisting of video-system, optoelectronic system and action-force measuring systems, was applied allowing a determination of the lumbar load of the healthcare worker very close to reality [15]. Skotte et al. [18] and also Schibye et al. [19] used a similar configuration to investigate the low-back load during common patient handling tasks. Additionally they measured muscle activity (EMG) in the Erector Spinae muscles and the degree of perceived exertion (RPE; Borg scale). These variables were not collected in the study described here, since the focus of our investigation laid solely on the determination of the lumbar load. Skotte's results - like in our study - revealed that compression force and torque showed a high task dependence whereas the EMG data and the questionnaire values were more dependent on the subject.

Another difference between the study described here and the investigations made by Skotte and Schibye concerns the used calculation model. While in the present case a 30-segment biomechanical model for the upper body parts (top down model) is used, both Skotte and Schibye underlayed their investigations a 7-segment model for the lower body parts (bottom up model). This "bottom up" calculation method, in combination with the derivation of the hand-action forces based on measuring the ground reaction forces, is considered less accurate for the calculation of spinal forces than the "top down" method, as comparative calculations with both approaches have shown [12].

Last but not least the activities investigated by Skotte et al. [18] related mainly to a subgroup of our scope of handlings (3 vs. 13) and, in part, to handlings combined with lower lumbar load (e.g. turning of the patient from lying on the back to lying on her side). Thus, other important activities, classified by the Statutory Accident and Health Insurance Institution for Health Services and Welfare Care (BGW) as definitely being endangering in the sense of the German occupational disease no. 2108 were not covered there.

Possible disadvantages of the whole measuring configuration used here originate from the limited spatial flexibility which restricts an application to the lab and makes an application to the clinical surroundings nearly impossible. On the other hand it is of positive relevance that with the lab measurements variables like postures, movements and forces of the healthcare worker, but also behaviour patterns of the patient which influence the lumbar load substantially can be controlled sufficiently.

Furthermore it could be marked critically that the sample is rather small. This was due to the complex measuring methods, which needed among other things, a huge amount of time for evaluation. To compensate for this constraint two professionally experienced healthcare workers acted as subjects, alternatively as patient or caregiver. They were both highly qualified in performing the tasks and in replicating different levels of patient's co-operatingness. The selected approach of the analysis of typical executions seems to be approved since exemplary evaluations of repeated measurements have shown a good correspondence of the results: 18 measurements for lifting a leg of a lying patient were accomplished and resulted in a mean value of 2.8 kN of compressive force with a range of 1.9 to 4.0 kN.

The inclusion of two experienced and trained physiotherapists acting as carer or patient could have led eventually to the fact that the activities were carried out in a too good manner in contrast to reality and it could therefore lead to an underestimation of the lumbar load in real environments (narcotised patient, restricted space in home care, two nurses simultaneous working, limited experience of novices). As a rule the investigations were arranged with partially co-operating patients. As mentioned before, in certain cases, however, handling was performed with a fully co-operating patient in order to protect the caregiver, hazarding again the consequence to underestimate the lumbar load in real environments.

A systematic overview especially to investigations with the main focus "patient transfer" is found with Hignett [20], Hignett et al. [21] and Hignett and Crumpton [22]. The authors took into consideration different transfer techniques and used aids as well as intervention options which should reduce the load of the healthcare workers. This aspect concerning the influence of different transfer techniques on the amount of the lumbar load was not taken into account in the present study, because the aim was to derive characteristic values of the lumbar load which can be used in occupational-disease statement procedures. Therefore only conventionally executed patient transfers, as normally performed in the daily clinical routine, were investigated, in order to avoid an underestimation of the lumbar load resulting from the use of load-reducing techniques which are not as common as advisable in Germany.

For the same reason this study does not even deal with the option of the use of lifters for patient transfers like Marras et al. [23] did. They stated that the use of ceiling-mounted patient lift systems leads to lumbar load that could be considered as safe, whereas floor-based patient handling systems had the potential to increase shear forces to unacceptable levels during manual patient handling.

Another interesting approach represents the investigation of Freitag et al. [24]. They state that awkward postures, even without manual object handling or patient transfers, may lead to a high risk of developing low back-pain. In their study they recorded all the postures and movements of nurses within a working shift. The results show that hundreds of stressful trunk postures occur in nursing work during a shift, and the authors concluded that preventive measures should not be restricted to manual patient handling due to the high exerted forces only, but should additionally consider tasks with awkward postures according to the high number of occurrence.

Other studies [5] do not focus on the quantitative determination of the lumbar load for short sequences as patient transfers like it is done in the present study. They describe the cumulative load over a working shift or a whole working life, based on the values of the present study. The resulting dose values can also give hints to the causes for the development of lumbar degenerative diseases, i.e. they investigate the dose-response relationship for the entire occupational life.

## Conclusions

The results of the study have shown that manual handling of patients - as it is routinely practised in hospitals or nursing homes - is associated with high lumbar load for healthcare workers and a considerable risk of developing intervertebral disc-related diseases must be taken into account. Prevention measures to avoid the appearance of lumbar overload are strongly needed. Future investigations should take various measures of the behavioural prevention by the application of "back-fairer" nursing techniques into consideration and also include the effect of applying technical aids (e.g. lifters) and small aids (e.g. sliding sheets) to reduce the lumbar load during manual patient handling. Quantitative measurements such as determined in this study, can help to evaluate the effectiveness of such preventive measures and to investigate dose-response relationships between the load on the lumbar spine and resulting diseases, to identify overload and - in the long run - to contribute to the reduction of low-back pain and musculoskeletal diseases.

## Competing interests

The authors declare that they have no competing interests.

## Authors' contributions

All authors designed and conducted the study. NW and STK initiated the study and configured the essentials for OD application. AL, MJ, NW and STK supervised the measurements. MJ and AL guided the evaluations and descriptions. CJ and AT developed the measuring devices and carried out data analysis. CJ prepared the manuscript. All authors have read and approved the manuscript.

## References

[B1] Mitchell T O'Sullivan PB Smith A Burnett AF Straker L Thornton J Rudd CJ Biopsychosocial factors are associated with low back pain in female nursing students: a cross-sectional study Int J Nurs Stud 2009 46 678 688 10.1016/j.ijnurstu.2008.11.004 19118828

[B2] Theilmeier A Jordan C Wortmann N Kuhn S Nienhaus A Luttmann A Jäger M Belastung der Lendenwirbelsäule von Pflegepersonen bei Patiententransfers - Kennwerte zur Nutzung in Berufskrankheiten-Feststellungsverfahren Zbl Arbeitsmed 2006 56 228 251

[B3] Kee D Seo SR Musculoskeletal disorders among nursing personnel in Korea Int J Indust Erogonomics 2007 37 207 212 10.1016/j.ergon.2006.10.020

[B4] Videman T Ojajärvi A Riihimäki H Troup JD Low back pain among nurses: a follow-up beginning at entry to the nursing school Spine 2005 30 2334 2341 10.1097/01.brs.0000182107.14355.ca 16227898

[B5] Seidler A Bergmann A Jäger M Ellegast R Ditchen D Elsner G Grifka J Haerting J Hofmann F Linhardt O Luttmann A Michaelis M Petereit-Haack G Schumann B Bolm-Audorff U Bolm-Audorff U Cumulative occupational lumbar load and lumbar disc disease - results of a German multi-center case-control study (EPILIFT) BMC Musculoskel Dis 2009 10 48 60 10.1186/1471-2474-10-48 PMC2689164 19422710

[B6] Bundesminister für Arbeit und Sozialordnung Zweite Verordnung zur Änderung der Berufskrankheitenverordnung Bundesgesetzblatt I 1992 59 2343 2344

[B7] Hartung E Schäfer K Jäger M Luttmann A Bolm-Audorff U Paul R Francks H-P Mainz-Dortmunder Dosismodell (MDD) zur Beurteilung der Belastung der Lendenwirbelsäule durch Heben oder Tragen schwerer Lasten oder durch Tätigkeiten in extremer Rumpfbeugehaltung bei Verdacht auf Berufskrankheit Nr. 2108. Teil 2: Vorschlag zur Beurteilung der arbeitstechnischen Voraussetzungen im Berufskrankheiten-Feststellungsverfahren Arbeitsmed Sozialmed Umweltmed 1999 34 112 122

[B8] Jäger M Luttmann A Bolm-Audorff U Schäfer K Hartung E Kuhn S Paul R Francks H-P Mainz-Dortmunder Dosismodell (MDD) zur Beurteilung der Belastung der Lendenwirbelsäule durch Heben oder Tragen schwerer Lasten oder durch Tätigkeiten in extremer Rumpfbeugehaltung bei Verdacht auf Berufskrankheit Nr. 2108. Teil 1: Retrospektive Belastungsermittlung für risikobehaftete Tätigkeitsfelder Arbeitsmed Sozialmed Umweltmed 1999 34 101 111

[B9] Kuhn S Baumann W Lang R Wortmann N MDD-Pflege - Vorläufige Dosisberechnung (Gesundheitsdienst) 2001 Berufsgenossenschaft für Gesundheitsdienst und Wohlfahrtspflege

[B10] Jäger M Theilmeier A Jordan C Luttmann A DOLLY Group Tartaglia R, Bagnara S, Bellandi T, Albolino S. Leiden: Taylor & Francis Biomechanical load on the lumbar spine for healthcare workers during patient transfer activities Healthcare Systems Ergonomics and Patient Safety 2005 365 369

[B11] Jäger M Luttmann A Göllner R Laurig W Society of Automotive Engineers: SAE The Dortmunder - Biomechanical model for quantification and assessment of the load on the lumbar spine Proceedings on the SAE Digital Human Modeling for Design and Engineeering Conference: 26-28 June 2001; Arlington 2001 2001-01-2085, 9 pp

[B12] Jäger M Belastung und Belastbarkeit der Lendenwirbelsäule im Berufsalltag - ein interdisziplinärer Ansatz für eine ergonomische Arbeitsgestaltung 2001 Fortschritt-Berichte VDI, Reihe 17, Nr. 208. Düsseldorf: VDI-Verlag

[B13] Jordan C Theilmeier A Luttmann A Jäger M Strasser H, Kluth K, Rausch H, Bubb H Optoelectronic posture recording during patient transfer for determining lumbar load Quality of Work and Products in Enterprises of the Future 2003 Stuttgart: Ergonomia-Verlag 1010 1013

[B14] Theilmeier A Jordan C Luttmann A Jäger M Messtechnisch gestützte Erfassung von Körperhaltungen und Aktionskräften zur Erhebung der Wirbelsäulenbelastung bei Pflegetätigkeiten Z Arbeitswiss 2005 59 162 171

[B15] Theilmeier A Jordan C Luttmann A Jäger M Measurement of action forces and posture to determine the lumbar load of healthcare workers during care-activities with patient transfers Ann of Occup Hyg 2010 54 923 933 10.1093/annhyg/meq063 20851849

[B16] Berufsgenossenschaft für Gesundheitsdienst und Wohlfahrtspflege BGW Belastungskataster BK 2108 1995 Stand 10/95 (unpublished manuscript). Mainz

[B17] Jordan C Theilmeier A Luttmann A Jäger M DOLLY Group Pikaar RN, Koningsveld EAP, Settels PJM Lumbar-load analysis for healthcare workers during patient transfer activities Meeting Diversity in Ergonomics Proceedings of the 16th World Congress on Ergonomics: 10-14 July 2006; Maastricht 2006 Amsterdam: Elsevier 6 pp on CD-ROM

[B18] Skotte J Essendrop M Faber Hansen A Schibye B A dynamic 3D biomechanical evaluation of the load on the low back during different patient handling tasks J Biomechanics 2002 35 1357 1366 10.1016/S0021-9290(02)00181-1 12231281

[B19] Schibye B Faber Hansen A Hye-Knudsen CT Essendrop M Böcher M Skotte J Biomechanical analysis of the effect of changing patient handling technique Appl Ergonomics 2003 34 115 123 10.1016/S0003-6870(03)00003-6 12628568

[B20] Hignett S Intervention strategies to reduce musculoskeletal injuries associated with handling patients: a systematic review [abstract] Occup Environ Med 2003 60 e6 10.1136/oem.60.9.e6 12937202 PMC1740617

[B21] Hignett S Crumpton E Ruszala S Alexander P Fray M Fletcher B Evidence-Based Patient Handling Tasks, Equipment and Interventions 2004 London: Routledge

[B22] Hignett S Crumpton E Competency based training for patient handling Appl Ergonomics 2007 38 7 17 10.1016/j.apergo.2006.02.004 16696933

[B23] Marras WS Knapik GG Ferguson SA Lumbar spine forces during manoeuvring of ceiling-based and floor-based patient transfer devices Ergonomics 2009 52 384 397 10.1080/00140130802376075 19296324

[B24] Freitag S Ellegast R Dulon M Nienhaus A Quantitative measurement of stressful trunk postures in nursing professions Ann Occup Hyg 2007 51 385 395 10.1093/annhyg/mem018 17715425

